# Supplementary material for: Pyrolysis Characteristics and Non-Isothermal Kinetics of Integrated Circuits
Source: Materials (Basel). 2022 Jun 24;15(13):4460. doi: 10.3390/ma15134460 (PMC9267151; doi:10.3390/ma15134460)
Supplement: Supplementary file 1 [file materials-15-04460-s001.zip › materials-1724134-supplementary.pdf]

Supplementary Material for "Pyrolysis characteristics and non-isothermal kinetics of integrated circuits"

Ziwei Chen[1,2], Linhao Liu[3], Hao Wang[4], Lili Liu[1,2,5] and Xidong Wang[1,2,5,*]

1 *Department of Energy and Resources Engineering, College of Engineering, Peking University, Beijing 100871, PR China; zwchen@pku.edu.cn (Z.C.); liu-0806@163.com (L.L.)*

2 *Beijing Key Laboratory for Solid Waste Utilization and Management, Peking University, Beijing 100871, PR China*

3 *Energy Bureau of Guangdong Province, Guangzhou 510030, PR China; 840159279@qq.com (L.L.)*

4 *School of Energy and Environmental Engineering, University of Science & Technology Beijing, Beijing 100083, PR China; wanghao3352@163.com (H.W.)*

5 *School of Materials Science and Engineering, Peking University, Beijing 100871, PR China*

**Correspondence: xidong@pku.edu.cn (X.W.)*

Table S1. Common reaction kinetics models and mechanism functions.

Code	Reaction model	Differential function $f(x)$	Integral function $F(x)$
A1	Nuclei production (n=1)	$1-x$	$-\ln(1-x)$
A2	Nuclei production (n=1.5)	$1.5(1-x)[-\ln(1-x)]^{1/3}$	$3(1-x)^{2/3}[1-(1-x)^{1/3}]$
A3	Nuclei production (n=2)	$2(1-x)[-\ln(1-x)]^{1/2}$	$[-\ln(1-x)]^{1/2}$
A4	Nuclei production (n=3)	$3(1-x)[-\ln(1-x)]^{2/3}$	$[-\ln(1-x)]^{1/3}$
A5	Nuclei production (n=4)	$4(1-x)[-\ln(1-x)]^{3/4}$	$[-\ln(1-x)]^{1/4}$
A6	Nuclei production (n=3/4)	$(3/4)(1-x)[-\ln(1-x)]^{1/4}$	$[-\ln(1-x)]^{3/4}$
C1	Phase boundary reaction (n=2)	$(1-x)^2$	$(1-x)^{-1}-1$
C2	Phase boundary reaction (n=3/2)	$2(1-x)^{3/2}$	$(1-x)^{-1/2}$
D1	Dimensional diffusion	$1/2x$	x^2
D2	Two-dimensional diffusion	$[-\ln(1-x)]^{-1}$	$x+(1-x)\ln(1-x)$
D3	Three-dimensional diffusion	$(3/2)[(1-x)^{-1/3}-1]^{-1}$	$[1-(2/3)x]-(1-x)^{2/3}$
D4	Three-dimensional diffusion	$(3/2)(1-x)^{2/3}[1-(1-x)^{2/3}]^{-1}$	$[1-(1-x)^{1/3}]^2$
D5	3-D diffusion (anti- Jander)	$(3/2)(1+x)^{2/3}[(1+x)^{1/3}-1]^{-1}$	$[(1+x)^{1/3}-1]^2$
D6	3-D diffusion (ZLT)	$(3/2)(1-x)^{4/3}[(1-x)^{-1/3}-1]^{-1}$	$[(1-x)^{-1/3}-1]^2$
D7	3-D diffusion (Jander)	$6(1-x)^{2/3}[1-(1-x)^{1/3}]^{1/2}$	$[1-(1-x)^{1/3}]^{1/2}$
D8	2-D diffusion (Jander)	$(1-x)^{1/2}[1-(1-x)^{1/2}]^{-1}$	$[1-(1-x)^{1/2}]^2$
R1	Shrinking core model	$2(1-x)^{1/2}$	$1-(1-x)^{1/2}$
R2	Shrinking core model	$3(1-x)^{2/3}$	$1-(1-x)^{1/3}$
R3	Shrinking core model (n=2)	$(1/2)(1-x)^{-1}$	$1-(1-x)^2$
R4	Shrinking core model (n=3)	$(1/3)(1-x)^{-2}$	$1-(1-x)^3$
R5	Shrinking core model (n=4)	$(1/4)(1-x)^{-3}$	$1-(1-x)^4$

Table S2. Comparison of activation energy obtained in this study with studies reported in literature.

Sample	Methods	Stream	E_a (kJ·mol^{-1})	Ref.
Electronic waste-non metallic fraction	CR	N_2	72-74	[S1]
Electronic waste-Printed circuit boards	Vyazovkin	N_2	93-308	[S2]
Electronic waste-Printed circuit boards	CR	N_2	149.28	[S3]
Electronic waste-Printed circuit boards	Friedman	N_2	86-193	[33]
Integrated circuits	Friedman, Kissinger, and CR	N_2	145-208	This study

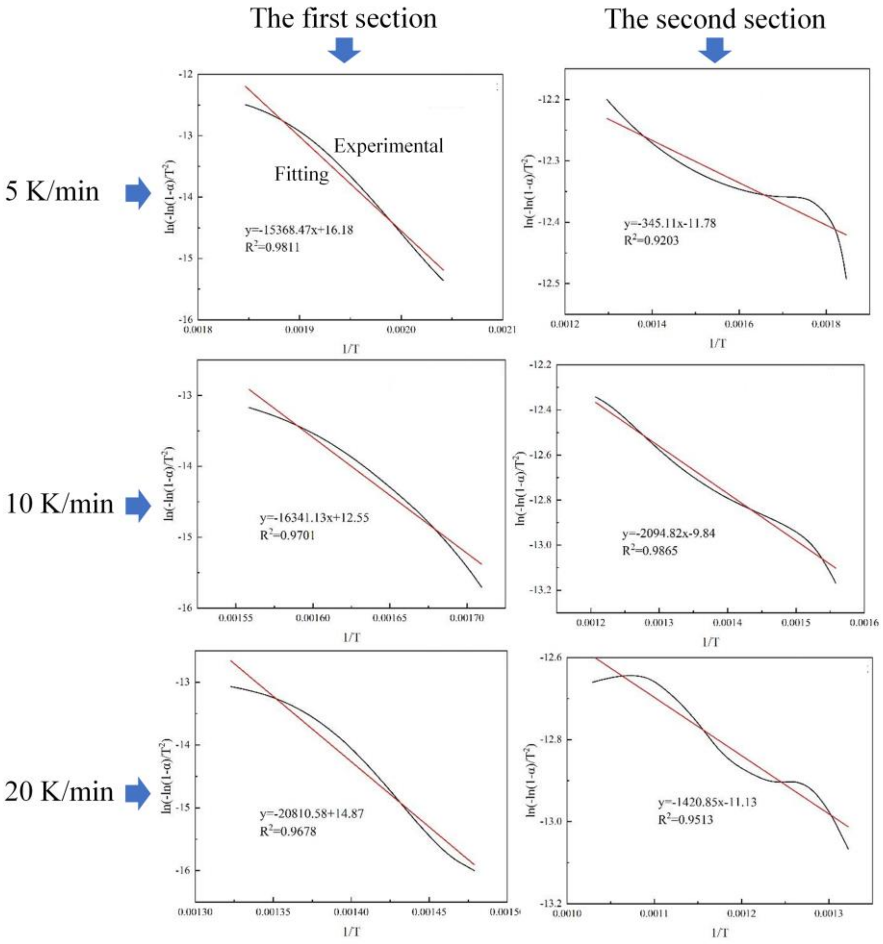

Figure S1. The linear regression for the extraction of the kinetic parameters from CR method.

References

[S1] Y. Shen, R. Yuan, X. Chen, X. Ge, M. Chen, Co-pyrolysis of E-waste nonmetallic residues with biowastes, ACS Sustain. Chem. Eng. 6 (2018) 9086–9093.

[S2] J.V.J. Krishna, S.S. Damir, R. Vinu, Pyrolysis of electronic waste and their mixtures: kinetic and pyrolysate composition studies, J.Environ. Chem. Eng. 9 (2021), 105382.

[S3] C. Quan, A. Li, N. Gao, Combustion and pyrolysis of electronic waste: thermogravimetric analysis and kinetic model, Procedia Environ. Sci. 18 (2013) 776–782.
